# Supplementary figures and images for: ‘We dry contaminated meat to make it safe’: An assessment of knowledge, attitude and practices on anthrax during an outbreak, Kisumu, Kenya, 2019
Source: PLoS One. 2021 Nov 4;16(11):e0259017. doi: 10.1371/journal.pone.0259017 (PMC8568283; doi:10.1371/journal.pone.0259017)

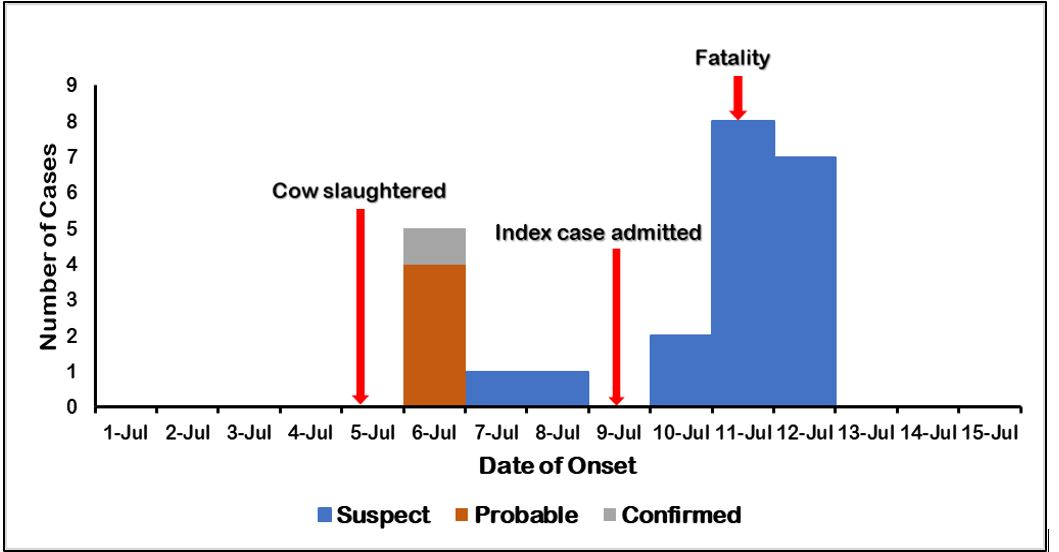

Supplement: S1 Fig — (TIF) [file pone.0259017.s001.tif]

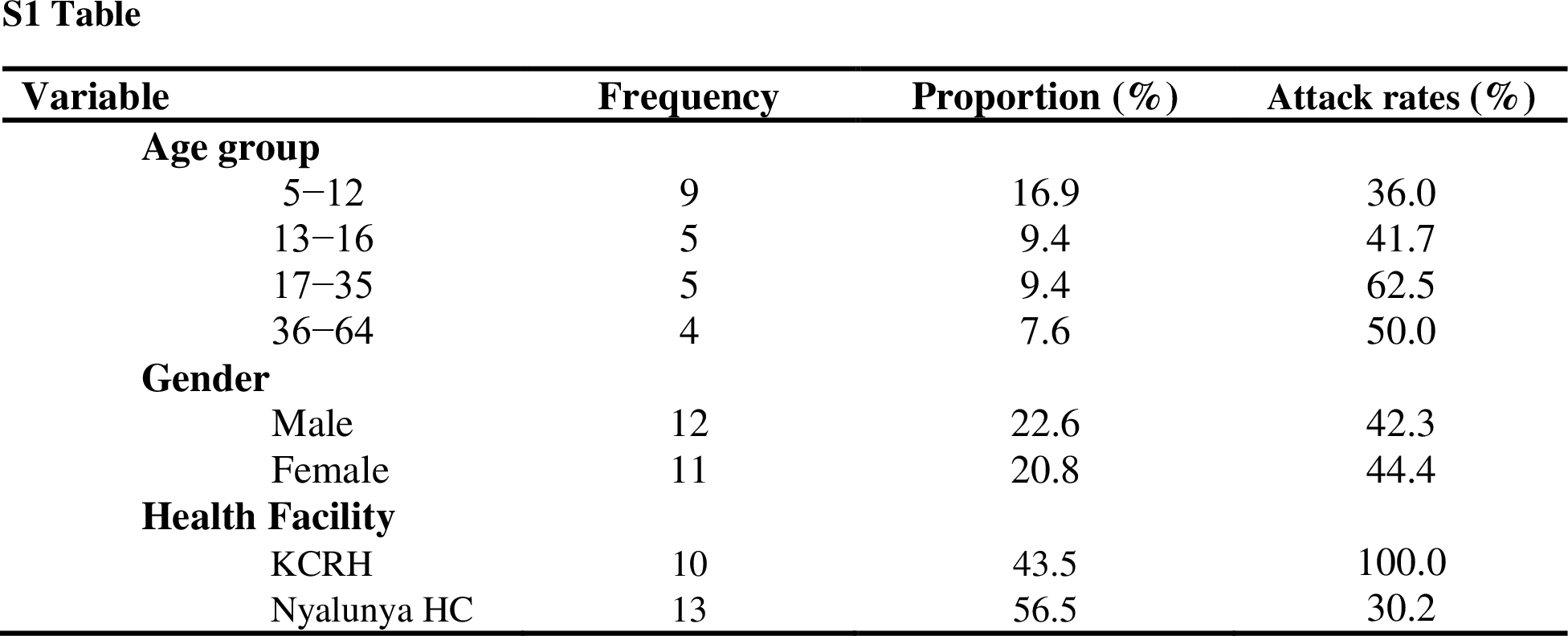

Supplement: S1 Table — (TIF) [file pone.0259017.s002.tif]

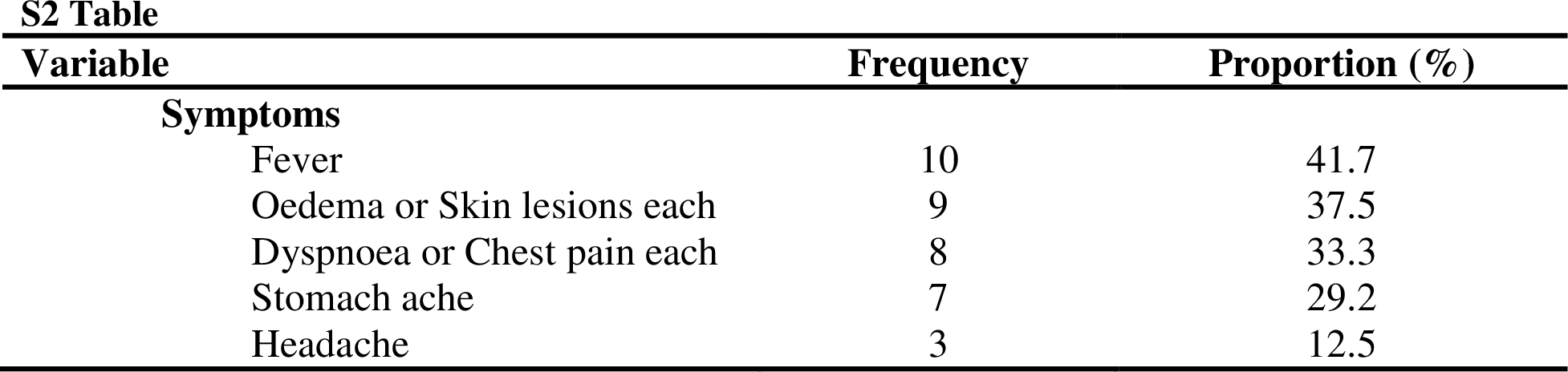

Supplement: S2 Table — (TIF) [file pone.0259017.s003.tif]

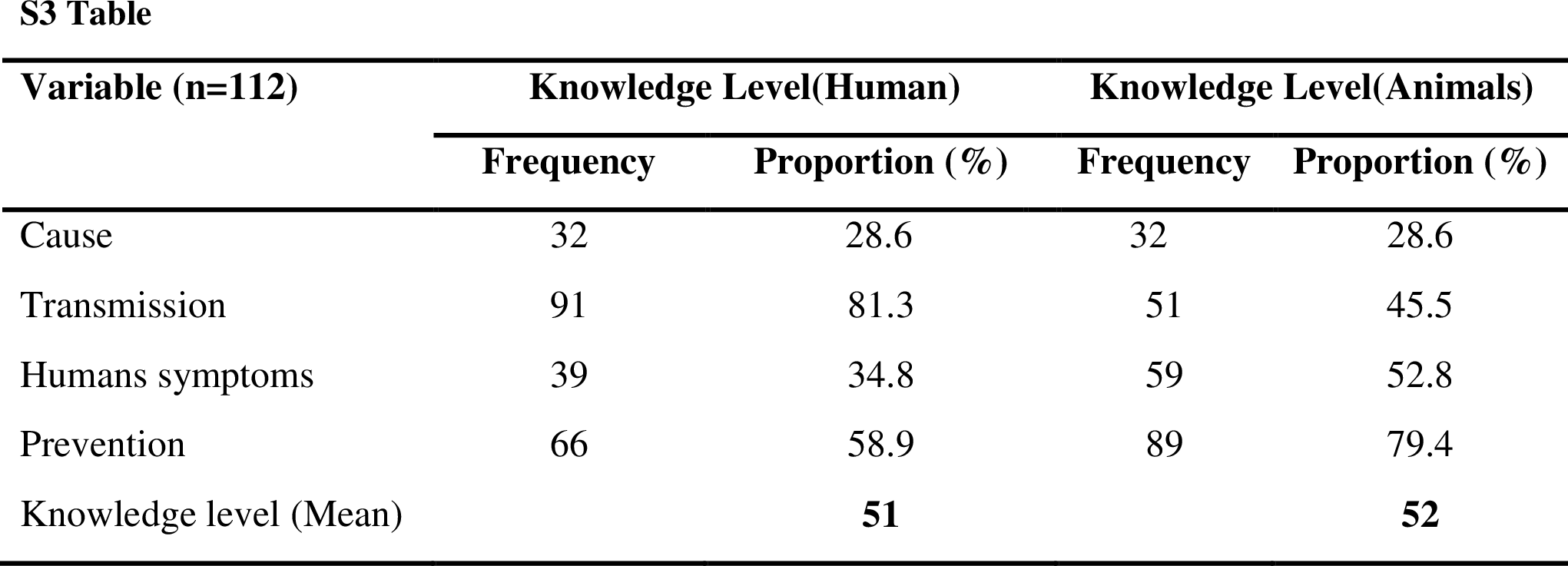

Supplement: S3 Table — (TIF) [file pone.0259017.s004.tif]

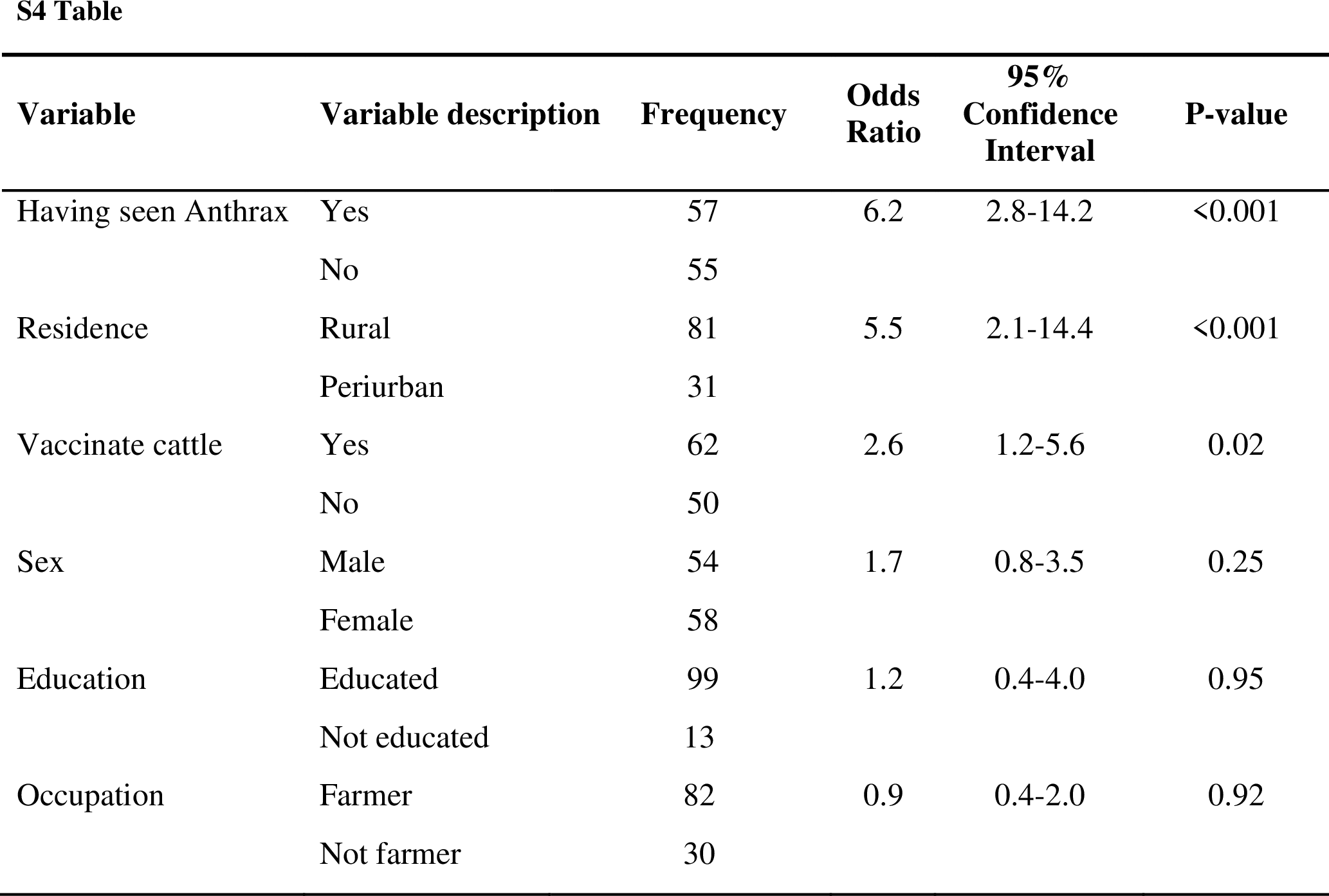

Supplement: S4 Table — (TIF) [file pone.0259017.s005.tif]

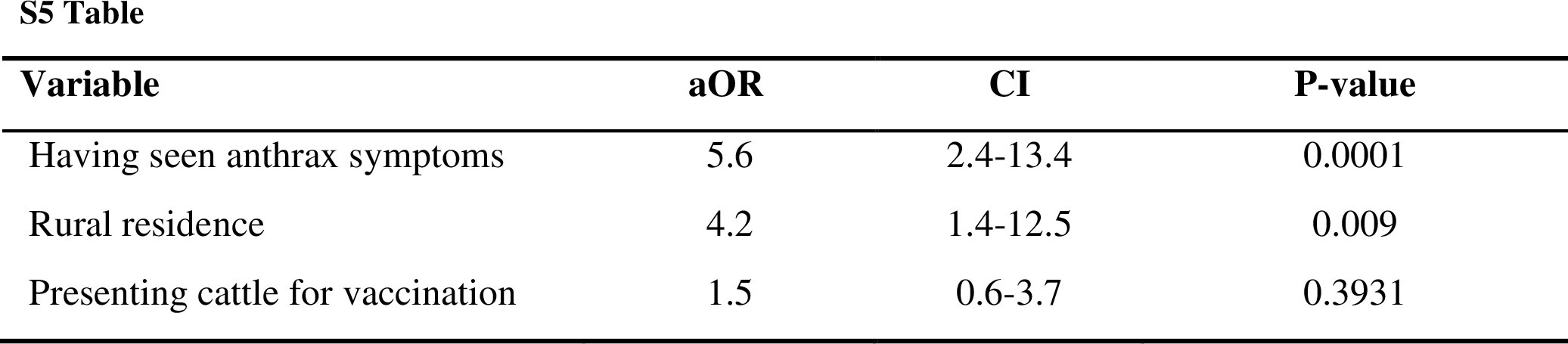

Supplement: S5 Table — (TIF) [file pone.0259017.s006.tif]
