# Supplementary material for: ‘We dry contaminated meat to make it safe’: An assessment of knowledge, attitude and practices on anthrax during an outbreak, Kisumu, Kenya, 2019
Source: PLoS One. 2021 Nov 4;16(11):e0259017. doi: 10.1371/journal.pone.0259017 (PMC8568283; doi:10.1371/journal.pone.0259017)
Supplement: S1 Appendix — (DOCX) [file pone.0259017.s007.docx]

**Appendices**

Household questionnaire

**Questionnaire for anthrax KAP study**

**Questionnaire No-----------------------**

**Date of Interview__________ Interviewer_____________**

**Sub County. _______________Ward _______________Village___________________**

**GPS Coordinates of Household: Longitude. _____________Latitude_____________**

Hello, my name is _______________. I am working with the Ministry of Health. We would like to ask you a few questions about anthrax in your community. This may take about 30-40 minutes.

| 1. What is your age in years? | 1. Gender | 1  0 | Male  Female |
| --- | --- | --- | --- |
|  |  |  |  |

**Background Socioeconomic & Education**

| 1. What is your highest level of education? |  | Primary  Secondary  College  Adult education  No formal education |
| --- | --- | --- |
| 1. Occupation |  | Formal employment  Farmer  Self employed  Casual  Other(specify) |
| 1. Which religion do you belong to? |  | Christian  Muslim  Hindu  Other (specify) |
| 1. Do you keep animals in your household? |  | Yes  No |
| 1. If yes, which animals do you keep (choose all that apply)   Cattle  Sheep  Goats  Pigs  Poultry  Donkey |  |  |
| 1. For how long have you kept animals   (round-up to the nearest whole number) |  |  |

**Anthrax General Knowledge**

| 1. Have you ever heard of an illness called Anthrax?   (if no, end the interview and thank the participant) |  | Yes  No/don’t know |
| --- | --- | --- |
| 1. Do you know what causes anthrax?   (if yes move to 11, if no/don’t know move to 12) |  | Yes  No/Don’t know |
| 1. If yes to 10, do you know what causes anthrax?   ***(Do not read. Check all that are mentioned.)*** |  | Germs  Witchcraft  Bad air  Eating meat from a dead animal  Skinning dead animal  Grazing animal in animal burial sites  other specify___________________ |
| 1. Which animals does it affect? |  | Cow  Goat  Sheep  Other (specify)_________________ |
| 1. Can you tell me how animals get infected with anthrax |  | Gazing in contaminated pasture  Drinking contaminated water  Grazing in animal burial sites  Don’t know |
| 1. How can you tell that an animal has died of anthrax? |  | Bleeding from mouth, nose, ears, anus  Rapid decomposition  Lack of rigor mortis  Bloating  Don’t Know  Others, specify___________________ |
| 1. Can anthrax be prevented in animals? |  | Yes  No  Don’t Know |
| 1. If Yes, How? |  | Vaccination  culling  environmental decontamination  Others(specify)  Don’t know |
| 1. When was the last time you vaccinated your animals against Anthrax? |  | <6 months  1 year  2 years  >3 yrs  Never |
| 1. Does anthrax affect humans?   (if no or don’t know skip to 28) |  | Yes  No/Don’t Know |
| 1. How do you think humans get infected with anthrax |  | Inhalation  Contact with infected tissues  Eating of meat  Others |
| 1. Can you tell me what the main symptoms of anthrax in humans are?   ***(Do not read. Check all that are mentioned.)*** |  | Skin lesions  severe diarrhea  severe cough  Other(specify)____________________  Don’t Know |
| 1. Can anthrax be prevented in humans |  | Yes  No/Don’t know |
| 1. If Yes,How?   ***(Do not read. Check all that are mentioned. Prompt after each response.)*** |  | Vaccination  Avoiding meat from sick animals |
| 1. Have you heard about the anthrax outbreak in your area recently? |  | Yes  No |
| 1. Have you heard of people in your village/neighborhood who died from anthrax in the past 1 year |  | Yes  No/Don’t Know |

**Attitude**

| 1. In your opinion, is anthrax a serious disease in animals in your area? |  | Yes  No/Don’t know |
| --- | --- | --- |
| 1. Who treats your animals when they get sick |  | Yourself  Another farmer  Local AHA  Vet  Do nothing |
| 1. In the course of treatment, what do you do when you realize that your animal will not survive? |  | Wait for it to die naturally and bury it  Slaughter it and burry  Slaughter it and sell the meat  Sell it to a butcher/trader  Slaughter it and give free meat to neighbors  Slaughter it, remove the skin and bury the carcass |
| 1. What will you do if your animal suddenly dies? |  | Slaughter it and sell the meat to neighbors  Sell the carcass to a butcher  Remove skin and burry  Remove skin and give meat to dogs  Call an animal service provider to conduct a post-mortem  Bury or burn the carcass |
| 1. What would you do if you or a member of your family had anthrax? |  | Seek treatment from a health facility  Use herbs  Buy drugs from a local chemist  Pray  Don’t Know |
| 1. What do you do if there is an anthrax outbreak in livestock in your area? |  | Call an animal service provider  Treat yourself  Do nothing  Use local medicine man  Others (specify)………………………. |

**Practices**

| 1. Have you ever eaten meat from animals that have died of unknown causes in the past one month? |  | Yes  No |
| --- | --- | --- |
| 1. Do you know of other people who have eaten meat from animals that have died of unknown causes? |  | Yes  No |
| 1. If Yes, Specify |  | Family  Neighbor  Friends |
| 1. Have you ever skinned an animal that has died suddenly in the past one year? |  | Yes  No |
| 1. Do you vaccinate your cattle against anthrax? |  | Yes  No/Don’t know |
| 1. If yes in 35 when did you last vaccinate your animal(s) |  | Less than 6 months  More than 6 months |
| 1. If No, Why? |  | Expensive  Time consuming  Vaccination site far from my home  Don’t know that am supposed to vaccinate  Other, specify_________________ |
| 1. Have you ever slaughtered an animal at home in the past one year? |  | Yes  No |
| 1. Was the meat inspected? |  | Yes  No |
| 1. If yes, who inspected the meat? |  | Animal health services provider  Public health officer  Headman  Butcher  Others |
| 1. Do you feel well informed about Anthrax? |  | Yes  No |
| 1. Do you wish you could get more information about Anthrax? |  | Yes  No |
| 1. What are the sources of information that you think can most effectively reach people like you with information on Anthrax? |  | Newspapers and magazines  Radio  TV  Billboards  Brochures,  posters and other printed materials Health workers  Religious leaders  Teachers  Other |
