# Supplementary material for: ‘We dry contaminated meat to make it safe’: An assessment of knowledge, attitude and practices on anthrax during an outbreak, Kisumu, Kenya, 2019
Source: PLoS One. 2021 Nov 4;16(11):e0259017. doi: 10.1371/journal.pone.0259017 (PMC8568283; doi:10.1371/journal.pone.0259017)
Supplement: Attachment — Submitted filename: PLOS ONE anthrax Review report.docx [file pone.0259017.s009.docx]

**Review report**

Summary:
In this study, one outbreak of anthrax in a single locality in western Kenya is described. The outbreak affected one cow and humans. The authors reviewed records for human and animal cases for the period from July 1 through July 28, 2019, conducted case search and contacts tracing using standard case definitions. They then conducted a cross-sectional study to assess community knowledge, attitude, and practices towards anthrax. The authors then discuss the importance of anthrax and the need for similar studies in similar scenarios.

However, knowledge is not expounded; only the summarized findings are provided. They are also not discussed at all. After reading the paper, it is difficult to describe what knowledge the community have. The only answer I have is that knowledge level above the mean was attained by 51 or 52% of the respondents

Attitude: ideally, there is a strong link between knowledge attitude and practices in a study. Indeed the assessment of knowledge is relevant only in so far as it influences attitude and practice. For instance in this study, for authors to study knowledge and attitude toward anthrax (both livestock and human) in the population, they should have first decided what facts about anthrax the population needs to be aware of in order to form certain attitudes. Unfortunately, the authors persistently claim that anthrax had not been reported in the area, yet “seeing anthrax” reached significance in its association with knowledge.

The study formulated a good research question to address a felt need. Indeed this target population selected itself since it experienced the outbreak. However, the however the preconceived idea that authors approached the area with, never reports of anthrax negates the purpose but instead should have been anchored on finding practices and attitudes on how sudden death among animals is perceived and viewed in the context of anthrax and also other zoonoses as well. It could have been relevant to include the nutritional, socio-economic pathways that could link livestock health and human welfare beyond Public health (PH).

This study should clearly highlight the following for it to be a strong KAP survey;

1. To identify and describe the baseline knowledge, myths, misconceptions, attitudes, beliefs, and behaviors in relation to both livestock and human anthrax
2. Provide information on need, issues, and barriers (all informed by KAP findings) related to the development of effective, locally relevant PH interventions

While this manuscript is a valuable contribution to our understanding of the occurrence and epidemiology and KAP of anthrax in endemic areas, significant modifications, language edits, significant change in references would be required prior to publication, as described in my major comments outlined below.

**Abstract**

- Line 54:Language edits; replace done with “carried out”
- Connect line 50& 51 for a clearer flow
- Language edits line 56 “was calculated”
- Language edits line 57 exposed “through”
- Language edits line 58 that begin with “Female ..is hanging. It is not complete
- Line 61: the knowledge level of 51% needs to be expressed in clear ways. It is knowledge per individual, or is it at the population level?
- Language edits line 62 “use a better term than “seen anthrax”
- Line 63: does the effect of vaccinating cattle apply to anthrax?
- Line 64: Regarding selling & slaughter- this is what they do whether the carcass is anthrax or not?
- Results in line 64-65 are confusing. Are they what they do or this is counterfactual?

Conclusion:

- Again, you may have meant to say that anthrax exposure was through consumption relative to other means of exposure. But your sentence could also be constructed to mean beef consumption as opposed to the consumption of other kinds of meat e.g mutton
- Poorly understood: Does your study equate knowledge and understanding

Results:

- Where are results describing attitude and practices?

**Manuscript Body**

- The opening sentence cant have suggested reference because there are better ones, this should be used in the context of either spatiotemporal clustering or community knowledge or anthrax
- Reference 2 is about malformed frogs in Minnesota?? Really suitable here?
- Reference 3 is a No when you have original research describing these processes very clearly
- Line 90 is incorrect; cutaneous form also has a range; it is not a definite one day IP
- Inhalation of anthrax can get delayed up to one or 2 months
- GI form is usually 2-5 days but could be as short as 15 hours
- The fact your sentences are not referenced is evidence that these could be speculations
- The “they” in line 92 is not clear whether it refers to human or livestock anthrax
- Line 95 is ambiguous. Individuals with cuts abrasion can only be at high risk of infection if handling infected carcasses
- Language edits line 95 “risk of ” not “risk to”
- Language edits line 96: the use of the word “like” doesn’t sound well
- Language edits line 97: how do animals that are resistant to anthrax spread or contaminate the environment?
- Reference 3 cannot provide a good reference for this sentence; look for Bengis and Frean 2014 and your reference 1
- The sentence in line 100 has been referenced with only 1 reference; these sentences require more references
- Line 102 is not correct; the seasonality of anthrax varies among locations making it difficult to develop a single consistent ecological description of anthrax
- Reference 9 should be Carlson et al 2019
- Line 107-110: Our recent research has found that up to 50 outbreaks have been reported, Because this work has not been published yet, mention that this could be higher due to underreporting
- Line 110: Muturi et al did not conduct the serology in 2017. Kindly insert the original reference that conducted the work
- Line 113: is not correct. Kisumu had reported anthrax in diverse dates between 1957 and 2017. The Evidence Is in the DVs records Kabete
- Line 114: How was the case confirmed; As far as I know confirming the human anthrax case is difficult; what samples were taken for example?
- Language edits
- Line 120: replace eaten with consumed
- Line 121: the suspect color; this cow hasn’t been & introduced for the word “the” to be used
- Line 124: depends on the specific area; village, ward, sub-county, or county. Be cautious as this might be corrects
  - The introduction focused more on what is known only to miss the following
  - It failed to justify and build the case for the study
  - Essential background information missed-KAP in other studies such as Sitali et al 2017, Chacha thesis UON, Mohamed et al 2019 in northern counties, etc
  - A significant sentence at the end also helps to frame the current status of the work in the field
- Line 130-131: None of the population resided in an urban area?
- Line 136: Here you describe the study as an outbreak investigation, any consideration for tilting it so; similar to Hassan et al 2020
- Line 138: Skin lesions could be specified as they are in human anthrax
- Line 141: the confirmed case here should be specified as livestock anthrax
- Line 138-143: this case definition should be clear for the reader that it pertains to human anthrax
- Line 146: change “a” case definition to “the” case definition
- Line 146-148: the non-specificity of human anthrax can not in many cases
- Line 150: add using a snowballing sampling technique
- Line 153: what “waste”?
- Line 156: Conducting KAP in one area you previously mentioned had not reported anthrax
- Line 161: it is nice to be transparent on how the sample size of 112 was determined.
- Line 171: that begins with… in the absence… is incomplete
- Line 173: it is not clear whose demographic data were collected
- Line 174:indicate that it is for anthrax that the cause, transmission, and identification information were sought
- Line 175: the author here mix up attitude and practices. While attitude refers to individual feeding towards a topical issue, plus any preconceived ideas that they may have towards it, practice refers to the ways in which they demonstrate their knowledge and attitude through their actions
- Line 195: replace “average” with “mean”
- Line 197 to 199: should be incorporated into line 60-61 to teach the reader early on, on how the study determined the knowledge level
- Line 221: it is important to describe transparently how the confirmed case was diagnosed including samples taken and plates/pictures of the diagnosis provided as previously it has been difficult to conclusively demonstrate bacteremia in resistant hosts such as human and carnivores
- Line 136: Spells the dates of outbreak investigation when signs and symptoms investigated were reported
- What date did the cow die and slaughtered?
- The reason for this chronology is the implication it has on confirmatory diagnosis
- With early cutaneous anthrax lesion, vesicular fluid in the untreated patient will yield *B. anthracis* on culture and likely reveal capsulated bacilli inappropriately stained smears. In older lesions, detection in smears or by culture requires lifting the edge of the eschar and obtaining the fluid. But the fluid will be sterile if the patient has been treated with an antibiotic which is more often the case
- Line 223:43.5% constitute the referrals: this is ok
- Line 224:43.4% is attack rate and Is ok
- Line 225: AR of 17% among those 5 and 12 years shouldn’t the denominator by the number of exposed persons in that age group? i.e better compute age-specific attack rates or sex-specific attack rates?
- Line 225: male attack rate of 22.6% denominator contains

1. All females
2. Males exposed but did not present disease

- Line 226: I guess the hand swelling you refer to is oedema. Consider referring it as such
- Tables 1 &2 show similar findings. They could be combined and preferably illustrate age and gender-specific attack rates
- Table 3 can be compressed to save on space e.g Hand oedema or skin lesion each 9% Dyspnoea or chest pain each 8%Nausea, Fatigue, and dizziness can be placed at the footnote and indicate that each had a frequency of 1
- My earlier concerns on confirmatory diagnosis are addressed in 238-9 with assuming that no antibiotics had prescribed prior to KCRH admission
- The information on lines 237-9 should come earlier on
- Findings between lines 238 and 241 are let as far as one health approach to control zoonoses is concerned. What were the responses from KCRH to the veterinary authorities? I expected to find this in the discussion
- Line 258: what mean do authors refer to? The 51% and 52%; if yes shouldn’t these be assessed by chi-square testing
- Much of 263-273 are in my view are practices
- Read the letter to the Editor by Sunil Raina to see what attitude is
- Line 292: is it “Aremo” or “Alemo”
- Table 6: is vaccinating cattle an independent factor given its P-Value?
- Line 309: Reinforces my earlier suggestion on the focus of this paper
- Line 309: the results are not explicit on the forms of anthrax encountered; this should be rectified
- Line 310: the authors are getting ambiguous and inconsistent; consuming beef i.e meat, or being exposed to a cow… what is defined exposed to a cow
- Line 311: was the study conducted to investigate the characteristics of a known outbreak of anthrax or conducted to investigate what was the outbreak about? Because if anthrax was known beforehand, line 311 is redundant, otherwise it is not!
- Line 313-315: connect the index case with the suspected cow first then branch out to the rest of the cases
- Line 316-317: I would be more cautious in asserting this sentence
- Line 318-320:Here the authors speculate, hypothesize, execute the hypothesis, and conclude in favor of their hypothesis linked to their findings. This could be inaccurate as well
- Line 324-325: I suggest the authors compute age-specific attack rates for more convincing evidence and generating a hypothesis
- Line 325: about pupils is too simplistic. Tighten the message
- The paragraph covering lines 321 to 333 is not well structured. Each paragraph must be
  - Unified meaning that all the sentences are related to one central point or idea i.e one paragraph one idea. This paragraph has 3 main ideas
  - Coherent meaning that sentences are logically organized and clearly connect. This is not possible without unification
  - Relevant meaning that the paragraph supports the overall theme and purpose of the paper
- Line 327: the one health approach needs to be specified in the context of this study e.g: Line 329-33 is an example of how this can be tied with lines 288-241
- Line 334: be cautious and mention that this was in wildlife, though
- The hypothesis put forward in lines 337-340 is not strong. The more plausible one is
  - Eating grass too close to the ground where spores may be found
  - Stress lowers the LD50
- Line 340: replace the word “never’
- Line 342-343: this cannot come up as a hypothesis when there were opportunities to ask; even later during the manuscript preparation; I guess even know if the household is contacted they would recall. i.e was this animal owner or for trade? Was it walking for long-distance? Tie with the stress mentioned above
- Line 346: low level of knowledge- connect with a “probable low incidence”
- Line 349-350: negates all the authors' statements about disease absence in the area
- Line 351-352: remove the word very
- Bleeding from the orifice is not characteristic
- Many animals die from and remain for hours without blood oozing from orifices
- Line 353: But there are a number of differential diagnoses of blood oozing from orifices especially hemorrhagic syndromes associated with RVF, *T. vivax*
- Line 353; which findings are referred to in this line?
- Line 355: is the surveillance in this line clinical?
- Line 360: Address the language in line 360..availability of cheap meat with poverty….
- Line 363: I didn’t find this result in the result section
- Line 367: Address the language in this line
- Line 369: could the same be said of anthrax?
- Line 376-377: these are not necessary as knowledge about the effect of physical treatments is available.. what is better to know is if there are untoward GI signs that consumers of that meat encounter?
