# Supplementary material for: ‘We dry contaminated meat to make it safe’: An assessment of knowledge, attitude and practices on anthrax during an outbreak, Kisumu, Kenya, 2019
Source: PLoS One. 2021 Nov 4;16(11):e0259017. doi: 10.1371/journal.pone.0259017 (PMC8568283; doi:10.1371/journal.pone.0259017)
Supplement: Attachment — Submitted filename: Anthrax Manuscript_Responce to Reviewers comments.docx [file pone.0259017.s010.docx]

To Joerg Heber

Editor-in-Chief

PLOS ONE

[1265 Battery Street, Suite 200](https://goo.gl/maps/SNG0D)
[San Francisco, CA 94111](https://goo.gl/maps/SNG0D)

[USA](https://goo.gl/maps/SNG0D)

Phone: +1(415) 624-1200
FAX: +1(415) 795-1584
[plos@plos.org](mailto:plos@plos.org)

29^th^ June, 2021

Dear Sir

Thank you for your reply regarding our manuscript number PONE-D-20-34615; titled
‘We dry contaminated meat to make it safe’: An Assessment of Knowledge, Attitude and Practices on Anthrax during an outbreak, Kisumu, Kenya, 2019’

We are grateful for your feedback, reviewer’s comments, and the positive evaluation of our work.

We have revised and modified the text and figures according to the reviewer’s critiques and suggestions.

Because of this, we provide new data information and explanations on the issues raised by the sections of the manuscript. We have also merged tables that had similar results and revised analysis of attack rates to age and gender specific attack rates. We now have five tables and not six.

We have also added many new and clarifying statements in all parts of the paper. These changes have improved the manuscript considerably and we hope that it can be considered for publication soon.

Sincerely,

Bernard Chege Mugo

Field Epidemiology and Laboratory Training Program,

Ministry of Health, Kenya

P. O. Box 225-00202,

Nairobi

**Detailed response: we have addressed editorial comments and responded to the comments by the reviewers as follows.**

**Response to Reviewers**

**Abstract**

**Reviewers Comment:** Line 61: the knowledge level of 51% needs to be expressed in clear ways. It is knowledge per individual, or is it at the population level?

**Author’s response:** This is knowledge at the population level not individual level.

**Reviewers Comment:** Line 63: does the effect of vaccinating cattle apply to anthrax?

**Author’s response:** This was in reference to the practice of presenting cattle for vaccination

**Reviewers Comment:** Line 64: Regarding selling & slaughter- this is what they do whether the carcass is anthrax or not?

**Author’s response:** Yes this is what they do to all the carcasses since at that level they are not able to find out if it is anthrax or not.

**Reviewers Comment:** Results in line 64-65 are confusing. Are they what they do or this is counterfactual?

**Author’s response:** This is factual derived form the responses during face to face interview

**Conclusion:**

**Reviewers Comment:** Poorly understood: Does your study equate knowledge and understanding

We have revised this statement; “The disease was poorly understood and this may hamper prevention and control measures” to “Low knowledge level on the disease may hamper prevention and control measures”. This is to remain focused on knowledge and not understanding.

**Results:**

**Reviewers Comment:** Where are results describing attitude and practices?

**Author’s response:** These are the summarized results for both attitude and practices

About 23.2% (26/112) would slaughter and sell meat to neighbors while 63.4% (71/112) would bury or burn carcass. Nearly 93.8% (105/112) believed vaccination prevents anthrax. However, 5.4% (62/112) present livestock for vaccination.**Manuscript Body**

**Reviewers Comment:** The opening sentence can’t have suggested reference because there are better ones, this should be used in the context of either spatiotemporal clustering or community knowledge or anthrax

**Author’s response:** We have removed the reference in the opening sentence

**Reviewers Comment:** Language edits line 97: how do animals that are resistant to anthrax spread or contaminate the environment?

**Author’s response:** Carnivores though resistant to anthrax can carry parts of infected carcass to other places acting as fomites and contaminating the environment.

**Reviewers Comment:** Reference 3 cannot provide a good reference for this sentence; look for Bengis and Frean 2014 and your reference 1

**Author’s response:** We have inserted this reference;

Bengis RG, Frean J. Anthrax as an example of the One Health concept. Rev Sci Tech. 2014;33: 593–604. doi:10.20506/rst.33.2.2309

**Reviewers Comment:** The sentence in line 100 has been referenced with only 1 reference; these sentences require more references

**Author’s response:**We have added more references as follows;

Goel AK. Anthrax: A disease of biowarfare and public health importance. World J Clin Cases. 2015;3: 20. doi:10.12998/wjcc.v3.i1.20

Mwakapeje ER, Høgset S, Softic A, Mghamba J, Nonga HE, Mdegela RH, et al. Risk factors for human cutaneous anthrax outbreaks in the hotspot districts of northern Tanzania: an unmatched case–control study. R Soc open Sci. 2018;5: 180479.

**Reviewers Comment:** Reference 9 should be Carlson et al 2019

**Author’s response:** We have put the reference as below;

Carlson CJ, Kracalik IT, Ross N, Alexander KA, Hugh-Jones ME, Fegan M, et al. The global distribution of bacillus anthracis and associated anthrax risk to humans, livestock and wildlife. Nat Microbiol. 2019;4:1337–43. https://doi.

**Reviewers Comment:** Line 110: Muturi et al did not conduct the serology in 2017. Kindly insert the original reference that conducted the work.

**Author’s response:** This is the correct reference;

Omballa VO, Musyoka RN, Vittor AY, Wamburu KB, Wachira CM, Waiboci LW, et al. Serologic evidence of the geographic distribution of bacterial zoonotic agents in Kenya, 2007. Am J Trop Med Hyg. 2016;94: 43–51. doi:10.4269/ajtmh.15-0320

**Reviewers Comment:** Line 124: depends on the specific area; village, ward, sub-county, or county. Be cautious as this might be corrects

- The introduction focused more on what is known only to miss the following
- It failed to justify and build the case for the study
- Essential background information missed-KAP in other studies such as Sitali et al 2017, Chacha thesis UON, Mohamed et al 2019 in northern counties, etc
- A significant sentence at the end also helps to frame the current status of the work in the field

**Author’s response:** This has been addressed towards the end of background section of the introduction.

**Reviewers Comment:** Reference 2 is about malformed frogs in Minnesota?? Really suitable here?

**Authors’ response:** We have replaced Reference 2 with the reference below;

Bengis RG, Frean J. Anthrax as an example of the One Health concept. Rev Sci Tech. 2014;33: 593–604. doi:10.20506/rst.33.2.2309

It now becomes reference 1 since we removed reference 1 in the opening sentence

**Reviewers Comment:** Reference 3 is a No when you have original research describing these processes very clearly.

**Authors’ response:** This reference

Ngetich W. Review of Anthrax: a Disease of Animals and Humans. Int J Agric Environ Bioresearch. 2019;4. Available: www.ijaeb.org

Was changed to;

Ravenel MP. Anthrax in Man and Animals. Am J Public Heal Nations Heal. 2008;30: 299–300. doi:10.2105/ajph.30.3.299

**Reviewers Comment:** Line 114: How was the case confirmed; As far as I know confirming the human anthrax case is difficult; what samples were taken for example?

**Authors’ response:** Confirmation was done by collecting whole blood sample and demonstration of *B. Anthracis* on a gram stained blood smear microscopic slide. The patient had not been treated with antibiotics.

**Reviewers Comment:** Line 121: the suspect cow; this cow hasn’t been & introduced for the word “the” to be used

**Authors’ response:** We have introduced the suspect cow during description of the index case and therefore the word “the” can be used.

**Reviewers Comment:** Line 130-131: None of the population resided in an urban area?

**Authors’ response:** The urban center is Kisumu town. The population of Kisumu East Sub county reside in Kisumu town periurban (Nyalenda and Manyatta wards). The rest of the wards are rural.

**Reviewers Comment:** Line 146-148: the non-specificity of human anthrax cannot in many cases

**Authors’ response:** We did not rely on the symptoms only we searched the patient’s history for information on whether the patients had a confirmed laboratory diagnosis, participated in the slaughter or consumption of beef from the suspected cow.

**Reviewers Comment:** Line 153: what “waste”?

**Authors’ response:** This is mainly ingesta from the gastrointestinal tract

**Reviewers Comment:** Line 156: Conducting KAP in one area you previously mentioned had not reported anthrax

**Authors’ response:** There has been reports of anthrax in Kisumu county at the director of veterinary services. However, these reports are not documented or published.

**Reviewers Comment:** Line 161: it is nice to be transparent on how the sample size of 112 was determined.

**Authors’ response:** We calculated sample size using Cochran formula (Israel, 2013) with 96% proportion for awareness(UON Repository) (Chacha at el 2017)

n = Z^2^* P (1-P)/d^2^

Where;

Z-value = 1.96 (Standard normal deviate for the 95% significance level)

p= Prevalence (96%) Proportion of people aware of anthrax

d = precision (0.05 at 95% significance level)

Therefore, n = {1.96^2^ * 0.96(1 – 0.96)/0.05^2^} = 56

Adjusting the sample size for design effect; 56*2 = 112 Households

**Reviewers Comment:** Line 173: it is not clear whose demographic data were collected

**Authors’ response:** Demographic information of the person being interviewed

**Reviewers Comment:** Line 221: it is important to describe transparently how the confirmed case was diagnosed including samples taken and plates/pictures of the diagnosis provided as previously it has been difficult to conclusively demonstrate bacteremia in resistant hosts such as human and carnivores

**Authors’ response:** Confirmation was done by collecting whole blood sample and demonstration of *B. Anthracis* on a gram stained blood smear microscopic slide. We were not able to get the plates and so we relied on medical reports at Kisumu County Referral Hospital.

**Reviewers Comment:** What date did the cow die and slaughtered?

**Authors’ response:** The cow was found dead in the morning of July 5, 2019 and slaughtered the same day.

**Reviewers Comment:** The reason for this chronology is the implication it has on confirmatory diagnosis

With early cutaneous anthrax lesion, vesicular fluid in the untreated patient will yield *B. anthracis* on culture and likely reveal capsulated bacilli inappropriately stained smears. In older lesions, detection in smears or by culture requires lifting the edge of the eschar and obtaining the fluid. But the fluid will be sterile if the patient has been treated with an antibiotic which is more often the case

**Authors’ response:** This was the chronology:

The verbal autopsy conducted by interviewing the window of the index case during active case search and contact tracing revealed the following information.

The problem begun on July 5, 2019 at around 6:30 am when her husband (index case) was called upon by the owner of the cow suspected to have died of anthrax to be engaged in the slaughter.

He left for the slaughter and came back at around 11:30 am carrying offals *(Matumbo)* with him. At the time of coming back the wife had left for job and so he decided to put the offals in the pot and cooked them by boiling as he waited for the wife to come back and fry the boiled offals for supper.

At supper time she fried the boiled offals *(Matumbo)*, served the husband and their first born son who was a grade one pupil at Nyalunya primary school. She did not eat the meal since she does not eat meat and their last-born daughter was asleep. On 7^th^ July 2019 the husband left for his casual job but on the evening of the same day he began complaining of intense pain and sores appeared on the right arm.

He was unable to get sleep the whole night due to pain. In the morning of 8^th^ July 2019 he was taken to a nearby private clinic, diagnosed and treated for allergic reaction with cetirizine and Ibuprofen for pain management. He was not treated with antibiotics.

The swelling had extensively spread to the face. His legs became very cold and swollen with fluid. The swelling extended to the right chest and neck even after treatment after which the wife decided to take him to Kisumu county referral hospital on 9^th^ July 2019, were he was admitted. He continued being treated for an allergic reaction until anthrax was confirmed by demonstration of *Bacillus anthracis* in blood smear made from a blood sample from him.

It was after this confirmation that the department of health conducted active case finding and more cases admitted at the referral hospital. She added that the cow was in very good body condition prompting villages to be tempted to make a meal out of it. Beef from the cow was sold to the villagers while pupils consumed at home during both lunch and supper.

**Reviewers Comment:** Line 225: AR of 17% among those 5 and 12 years shouldn’t the denominator by the number of exposed persons in that age group? i.e better compute age-specific attack rates or sex-specific attack rates?

**Authors’ response:** We have revised these results to present the age group specific and sex specific attack rates as in the table below;

| **Variable** | **Frequency of cases** | **At risk** | **Proportion (%)** |
| --- | --- | --- | --- |
| **Age group** |  |  |  |
| 5−12 | 9 | 25 | 36.0 (9/25) |
| 13−16 | 5 | 12 | 41.7 (5/12) |
| 17−35 | 5 | 8 | 62.5 (5/8) |
| 36−64 | 4 | 8 | 50.0 (4/8) |
| **Gender** |  |  |  |
| Male | 12 | 27 | 44.4 (12/27) |
| Female | 11 | 26 | 42.3 (11/26) |

**Reviewers Comment:** Tables 1 &2 show similar findings. They could be combined and preferably illustrate age and gender-specific attack rates

**Authors’ response:** We have combined table 1 and 2 and included both frequency distribution, age and gender-specific attack rates. Since frequency distribution is described in the results section as narrative, I found it advisable to retain it in the table for reference purposes.

**Reviewers Comment:** Table 3 can be compressed to save on space e.g Hand oedema or skin lesion each 9% Dyspnoea or chest pain each 8%Nausea, Fatigue, and dizziness can be placed at the footnote and indicate that each had a frequency of 1

**Authors’ response:** Table 3 has been revised as below and labeled table 2 since we combined table 1 and 2.

| **Table 2 Distribution of Symptoms of Anthrax, Kisumu East Sub county 2019. (n=23)** | | |
| --- | --- | --- |
| **Variable** | **Frequency** | **Proportion (%)** |
| **Symptoms** |  |  |
| Fever | 10 | 41.7 |
| Oedema or Skin lesions each | 9 | 37.5 |
| Dyspnoea or Chest pain each | 8 | 33.3 |
| Stomach ache | 7 | 29.2 |
| Headache | 3 | 12.5 |

Nausea, Fatigue and Dizziness each had a frequency of 1

**Reviewers Comment:** My earlier concerns on confirmatory diagnosis are addressed in 238-9 with assuming that no antibiotics had prescribed prior to KCRH admission.

**Authors’ response:** We confirm that no antibiotics were prescrided since the patient was being treated for an allergic reaction prior to referral to KCRH. He was put on Cetirizine (antihistamine) and Ibuprofen (Anti-inflammatory and analgesic)

**Reviewers Comment:** The information on lines 237-9 should come earlier on

**Authors’ response:** We have shifted this information to the background section

**Reviewers Comment:** Line 258: what mean do authors refer to? The 51% and 52%; if yes shouldn’t these be assessed by chi-square testing

**Authors’ response:** During analysis, we calculated the two means ie the 51% and 52%; In conducting these statistics, we wanted to find out if there exists statistically significant difference in the two means and the most appropriate test for the difference in the means is a paired t-test in this case as opposed to a chi-square test.

**Reviewers Comment:** Table 6: is vaccinating cattle an independent factor given its P-Value?

**Authors’ response:** By this, we meant to consider the practice of farmers taking willingly taking their animals to the crush to have their cattle vaccinated. We wanted to assess whether there is a difference in this practice between those who have knowledge on anthrax and those who do not have. We wanted to find out if willingness to have animals vaccinated against anthrax is driven by having knowledge on the disease or not.

**Reviewers Comment:** Line 309: the results are not explicit on the forms of anthrax encountered; this should be rectified.

**Authors’ response:** This has been revised as follows;

The presenting clinical symptoms including oedema and skin lesions are evidence for cutaneous form of anthrax. Stomach ache and nausea are indicative of gastrointestinal form while the rest of the symptoms are as a result of systemic involvement most likely associated with consumption of the suspicious beef pointing to gastrointestinal form.

**Reviewers Comment:** Line 311: was the study conducted to investigate the characteristics of a known outbreak of anthrax or conducted to investigate what was the outbreak about? Because if anthrax was known beforehand, line 311 is redundant, otherwise it is not!

**Authors’ response:** By the time of conducting this study, a diagnosis of anthrax had been made and so we were investigating characteristics of a known outbreak of anthrax. I had put this stamen to strengthen the fact that most of our findings are consistent with what is expected on anthrax. However, for this reason I drop line 311.

**Reviewers Comment:** Line 318-320:Here the authors speculate, hypothesize, execute the hypothesis, and conclude in favor of their hypothesis linked to their findings. This could be inaccurate as well.

**Authors’ response:** This has been corrected

**Reviewers Comment:** Line 324-325: I suggest the authors compute age-specific attack rates for more convincing evidence and generating a hypothesis

**Authors’ response:** We computed age-specific attack rates and found out that it was highest among persons aged between 17 to 35 years, and not 5 to 12 years.

**Reviewers Comment:** Line 325: about pupils is too simplistic. Tighten the message

**Authors’ response:** The hypothesis has changed with the change in attack rate as above

**Reviewers Comment:** The paragraph covering lines 321 to 333 is not well structured.

**Authors’ response:** This mentioned paragraph has been revised to reflect the reviewers comments

**Reviewers Comment:** The hypothesis put forward in lines 337-340 is not strong. The more plausible one is

- Eating grass too close to the ground where spores may be found
- Stress lowers the LD50

**Authors’ response:** Since anthrax is soil-borne, the occurrence of outbreaks at the beginning of a dry season could be attributed reduced pastures resulting to herbivores eating grass too close to the ground where spores may be found. Stress factors associated with dry seasons including poor nutrition lowers LD_50_ for *B. Anthracis* toxins increasing susceptibility to infection.

**Reviewers Comment:** Line 327: the one health approach needs to be specified in the context of this study e.g: Line 329-33 is an example of how this can be tied with lines 288-241

**Authors’ response:** Reviewers concern above has been addressed

**Reviewers Comment:** Line 355: is the surveillance in this line clinical?

**Authors’ response:** By community engagement, we meant using participatory approach techniques like participatory disease surveillance to utilize existing ethno veterinary knowledge in the community for surveillance of livestock diseases. This will improved reporting, and early detection of diseases as compared to passive surveillance alone. I had been realized during household interviews that members of the community have a lot of ethno veterinary knowledge on livestock health.

**Reviewers Comment:** Line 376-377: these are not necessary, as knowledge about the effect of physical treatments is available. What is better to know is if there are untoward GI signs that consumers of that meat encounter?

**Authors’ response:** Additional studies could elucidate on any unanticipated gastrointestinal signs associated with consumption of such dried meat depending on the mode and duration of drying.
