# Supplementary material for: ‘We dry contaminated meat to make it safe’: An assessment of knowledge, attitude and practices on anthrax during an outbreak, Kisumu, Kenya, 2019
Source: PLoS One. 2021 Nov 4;16(11):e0259017. doi: 10.1371/journal.pone.0259017 (PMC8568283; doi:10.1371/journal.pone.0259017)
Supplement: Attachment — Submitted filename: Response to Reviewers_3.docx [file pone.0259017.s011.docx]

To Joerg Heber

Editor- in-Chief

PLOS ONE

1265 Battery Street, Suite 200

San Francisco, CA 94111

USA

Phone: +1(415) 624-1200

FAX: +1(415) 795-1584

2^nd^ October, 2021

Dear Sir

Thank you for your reply regarding our manuscript number PONE-D-20-34615R1 of 28^th^ July, 2021; titled ‘We dry contaminated meat to make it safe’: An Assessment of Knowledge, Attitude and Practices on Anthrax during an outbreak, Kisumu, Kenya, 2019’.

We are grateful for your feedback, reviewer’s comments, and the positive evaluation of our work. We have revised and worked on the reviewer’s critics and suggestions mainly copyediting which was main suggestion by the reviewers.

During copyediting, we used an online software called Grammarly which helped us to identify grammatical errors including punctuation, spacing between words, use of articles and the general sentence structure. This will be viewed in the manuscript document in track changes.

We believe that the changes we have done will improve the manuscript considerably and we hope that it can be considered for publication soon.

Sincerely,

Bernard Chege Mugo

Field Epidemiology and Laboratory Training Program,

Ministry of Health, Kenya

P.O. Box 225-00202

Nairobi

**Detailed response: we have addressed copyediting comments and responded to the comments by the reviewers as follows.**

**Response to Reviewers**

Line 53; Article ‘the’ to read The household anthrax.

Line 54 putting article ‘the’

Line 60 Article ‘the’

Line 61 Article ‘the’ and adding ‘was’

Line 63 Article ‘the’knowledge’

Line 69 Article ‘the’

Line 72 ‘were’ instead of ‘was’

Line 126 ‘demonstrating’ instead of ‘demonstration’

Line 142 ‘Before’ instead of ‘prior’

Line 218 ‘were’ instead of ‘was’

Line 275 Article ‘the’

Line 277 inserted ‘with the’

Line 301 article ‘the’

Line 305 replaced ‘aged’ with ‘elderly ‘

Line 350 removed ‘having’

Line 351 removed ‘only’

Line 370 replaced ‘usually’ with ‘which is’

Line 378 inserted article ‘the’

Line 383 inserted article ‘the’

Line 384 inserted article ‘the’

Line 386 revised changing ‘sort’ to ‘sought’

Line 409, 410 and 411 revised to capture more details on previous anthrax outbreaks in Kisumu

Line 418 inserted article ‘The’

Line 429 inserted article ‘The’ The existence

Line 434 replaced ‘food borne’ with ‘food-borne’

Line 441 inserted article ‘The’

Line 445 inserted article ‘The’

The limitations section was revised to add the confirmatory diagnosis was done in line with guidelines

Line 480 replaced ‘concerning’ with ‘in relation’
